# Effect of target gene sequence evenness and dominance on real-time PCR quantification of artificial sulfate-reducing microbial communities

**DOI:** 10.1371/journal.pone.0299930

**Published:** 2024-03-07

**Authors:** Zhe Du, Sebastian F. Behrens

**Affiliations:** 1 Center for Environmental Health Risk Assessment and Research, Chinese Research Academy of Environmental Sciences, Beijing, China; 2 The BioTechnology Institute, University of Minnesota Twin Cities, St. Paul, Minnesota, United States of America; 3 Department of Civil, Environmental, and Geo-Engineering, University of Minnesota Twin Cities, Minneapolis, Minnesota, United States of America; Fudan University, CHINA

## Abstract

Quantitative real-time PCR of phylogenetic and functional marker genes is among the most commonly used techniques to quantify the abundance of microbial taxa in environmental samples. However, in most environmental applications, the approach is a rough assessment of population abundance rather than an exact absolute quantification method because of PCR-based estimation biases caused by multiple factors. Previous studies on these technical issues have focused on primer or template sequence features or PCR reaction conditions. However, how target gene sequence characteristics (e.g., evenness and dominance) in environmental samples affect qPCR quantifications has not been well studied. Here, we compared three primer sets targeting the beta subunit of the dissimilatory sulfite reductase (*dsrB*) to investigate qPCR quantification performance under different target gene sequence evenness and dominance conditions using artificial gBlock template mixtures designed accordingly. Our results suggested that the qPCR quantification performance of all tested primer sets was determined by the comprehensive effect of the target gene sequence evenness and dominance in environmental samples. Generally, highly degenerate primer sets have equivalent or better qPCR quantification results than a more target-specific primer set. Low template concentration in this study (~10^5^ copies/L) will exaggerate the qPCR quantification results difference among tested primer sets. Improvements to the accuracy and reproducibility of qPCR assays for gene copy number quantification in environmental microbiology and microbial ecology studies should be based on prior knowledge of target gene sequence information acquired by metagenomic analysis or other approaches, careful selection of primer sets, and proper reaction conditions optimization.

## Introduction

Quantitative real-time PCR (qPCR) of phylogenetic and functional marker genes is among the most commonly used techniques to quantify the abundance of microbial taxa in environmental samples [1] However, in most environmental applications, qPCR is regarded more as a rough estimation of population abundance than an exact absolute quantification method, because of quantification biases to the original target gene abundance produced during qPCR amplification for diverse template mixtures. Several potential causes of such biases due to primer or template sequence features have been described in the literature. For example, applying universal (degenerated) primers leads to discrimination during PCR amplification [2]. Primer sequence mismatch (MM) and MM location are also considered sources of amplification biases [3]. Differences in template sequence features such as GC-content can also cause discrimination during amplification [1, 4–6]. Other potential causes of biases during PCR amplification have been reported to be associated with PCR reaction conditions, such as different DNA polymerase amplification efficiencies, varying annealing temperatures, amplicon length, PCR cycle number, and DNA template concentration (i.e., low DNA template concentration might negatively affect the detection and accurate quantification of target genes in environmental samples) [7–10]. In recent years, biases of PCR-based techniques caused by low biomass samples received growing attention, especially for next-generation sequencing analysis. Low template concentration may introduce significant background noises into the sequencing results [11, 12]. However, its effect on qPCR quantification analysis hasn’t been thoroughly assessed.

The microbial world has been shown to hold tremendous diversity, and microbial communities are complex entities with varying taxa composition, richness, evenness, and dominance. Despite identifying numerous potential causes described above for PCR biases during the amplification process, the microbial community characteristics, specifically the target gene sequence features, could also contribute to PCR amplification biases and affect quantification results [9, 13]. For example, the target gene sequence richness was described as problematic when quantifying the abundance of microbial taxa in environmental DNA extracts [14]. However, it has not been documented in great detail how target gene sequence evenness and dominance affect qPCR quantification performance. It is also not well known for environmental applications of qPCR to what extent the use of degenerated primers affects absolute quantification results.

In this study, we compared three primer sets with different levels of degeneracy targeting the beta-subunit of the dissimilatory sulfite reductase (*dsrB*) gene to analyze how target gene sequence evenness (i.e., relative sequence abundance distribution) and dominance (i.e., the identity of dominant sequences) affect qPCR quantification results under two template concentration conditions (~10^8^ copies/μL and ~10^5^ copies/μL, defined as high and low template concentration conditions), in order to address two hypotheses. First, we hypothesized that target gene sequence evenness and dominance in environmental samples affect absolute qPCR amplification results. Second, we hypothesize that primer choice can help to minimize such effect on qPCR results as long as primer sequences are carefully selected or tuned to template sequence composition. Our reasoning to test the two template concentration conditions is as follows. The *dsrB* gene concentration in DNA extracts from sulfate reducer-enriched environments or pure cultures could reach a similar level as the high template concentration condition above [15]. Many other environmental systems could have a *dsrB* gene concentration around a low template concentration level after going through the standard DNA extraction protocol [16]. The three primer sets with different levels of degeneracy were chosen based on their various in silico coverage of *dsrB* gene sequences in a newly published database [17]. All evaluation experiments were performed using artificially assembled mixtures of different *dsrB* gene sequences (gBlock template mixtures). We varied the *dsrB* gene sequence evenness and dominance to construct these gBlock template mixtures (gBTMs) under each template concentration condition. To reduce potential quantification biases from PCR reaction conditions and ensure assay comparability, we used a standard curve-based method (Cy0) for qPCR data analysis [18]. The Cy0 algorithm does not require the assumption of uniform reaction efficiency between standards and artificial gBTMs or require any prior choice of fluorescence threshold level. Our results provide experimental evidence of how target gene sequence evenness and dominance at different template concentration conditions affect qPCR quantification results. We also evaluated how degenerate primer sets work on absolute quantification of target gene sequences in qPCR assays.

## Materials and methods

### Primer sequences

The *dsrB* gene primer sets DSRp2060F/DSR4R, DSR1728Fmix/DSR4Rmix, and DSR1762Fmix/DSR2107Rmix were synthesized by the University of Minnesota Genomic Center (UMGC). Forward and reverse primer degeneracies were 4/1, 77/10, and 98/29, respectively (S1 Table in S1 File). Primer degeneracy indicates how many oligonucleotide sequences comprise each primer mix [2]. S1 Table in S1 File lists primer amplicon length and binding sites in reference to the *dsrAB* gene sequence from *Desulfovibrio vulgaris* Hildenborough, a well-studied model microorganism among sulfate-reducing bacteria. Geneious 10.0.5 (Biomatters Ltd., Auckland, New Zealand) was used to evaluate the in silico coverage of each forward and reverse primer using a recently published *dsrAB* gene sequence database [19] with primer binding site searching criteria of 0 or 1 MM (S1 Table in S1 File).

### Assay optimization and qPCR conditions

All qPCR experiments for assay optimization were performed in triplicates on a CFX Connect^™^ Real-Time PCR Detection system (Bio-Rad, Hercules, CA, USA). The reaction volume was 20 μL, consisting of 1-fold SsoFast^™^ EvaGreen^®^ Supermix with Low Rox (Bio-Rad, Hercules, CA, USA), 0.5 μg/μL bovine serum albumin (BSA) (Roche Diagnostics, Indianapolis, IN, USA), forward and reverse primers at final concentrations of 400 nM/300 nM, 2 μM/2.1 μM (400 nM × 5/300 nM × 7), and 4μM/2 μM (400 nM × 10/400 nM × 5) for DSRp2060F/DSR4R, DSR1728Fmix/DSR4Rmix, and DSR1762Fmix/DSR2107Rmix, respectively, and molecular biology grade water (Sigma-Aldrich, St. Louis, MO, USA) (S2 Table in S1 File). Optimal primer concentrations for each primer set were empirically determined in a set of qPCR experiments by comparing the C_T_ values under different primer concentration conditions using a 500 bp fragment of the *dsrB* gene from *Desulfovibrio vulgaris* Hildenborough (0.01 ng/μL) as the template (S3 Table in S1 File). Thermocycling protocols were the same for all three primer sets except for slight differences in annealing temperature: initial denaturation at 95°*C* for 5 min, followed by 35 cycles of 95°*C* for 30 s, annealing at 58/55/55°*C* (DSRp2060F/DSR4R, DSR1728Fmix/DSR4Rmix, and DSR1762Fmix/DSR2107Rmix, respectively) for 30 s, and 72°*C* for 45 s (S2 Table in S1 File). The optimal annealing temperature for each primer set was also determined empirically by gradient qPCR using the same *dsrB* gene fragment from *Desulfovibrio vulgaris* Hildenborough (0.01 ng/μL) (S4 Table in S1 File), which was used as a standard DNA template for generating all following qPCR standard curves.

### qPCR standards, Cy0, and absolute *dsrB* gene copy quantification

A 10-fold standard dilution series was prepared, ranging in concentration from 10^−7^ ng/μL to 0.1 ng/μL. According to Eq 1, this corresponds to 2.34 × 10^2^ gene copies /μL to 2.34 × 10^8^ gene copies/μL.


$$number\;of\;copies\left(molecules\right)=\frac{X\;ng\times N_{A}}{\left(L\times 660\frac{g}{mole}\right)\times 1\times 10^{9}\;ng/g}$$ (1)

X is the number of templates in each PCR reaction. N_A_ is the Avogadro constant (6.022 × 10^23^ molecules/mole), while L is the length of the PCR product (390 bp).

The Cy0 of each qPCR reaction was calculated using the ‘qpcR’ package in R [20]. The Cy0 is the intersection point of the abscissa axis with the tangent of the inflection point of the Richards curve, which is obtained by non-linear regression fitting of the raw fluorescence data to a 5-parameter Richards function. A more detailed description of the Cy0 algorithm can be found in its original study [18]. Cy0 values and the log of the *dsrB* gene copy numbers from the standard dilution series were used to generate qPCR standard curves (S1 Fig in S1 File). Linear regression was then used to quantify the absolute target gene copy numbers of all the samples in this study. Standard curves of all three qPCR assays with different *dsrB* gene primers had R^2^ values > 0.99 (S1 Fig in S1 File). The more target-specific primers DSRp2060F/DSR4R showed a slightly more extensive detection range, with an absolute detection limit of 10^2^ gene copies/μL (S1 Fig in S1 File). In contrast, the qPCR assays of highly degenerate primer sets had an absolute detection limit of 10^3^ gene copies/μL.

### gBTMs preparation

#### *dsrB* gene gBlock sequences selection

**Fig 1:**
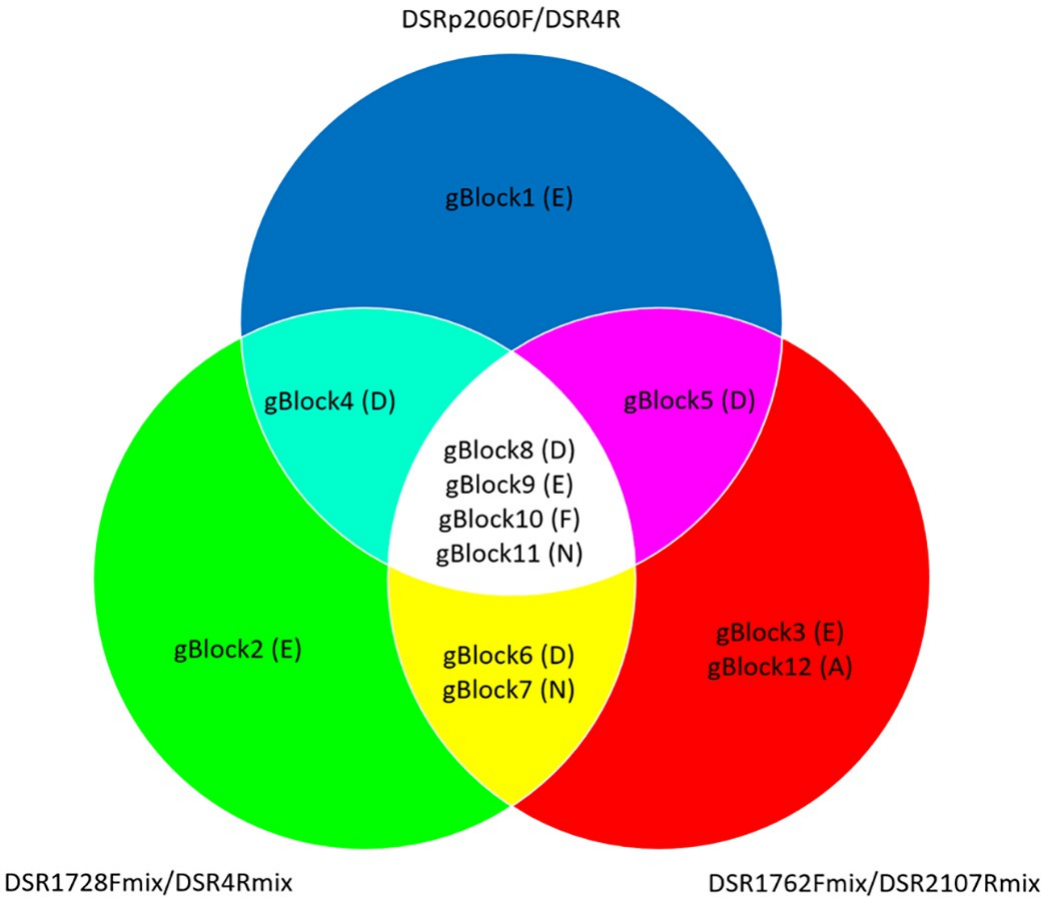
*dsrB* gBlocks and their primer-binding sites. The 12 gBlocks were selected to cover the currently known phylogenetic sequence diversity of *dsrAB* genes from sulfate-reducing bacteria and archaea. The five *dsrAB* gene phylogenetic superclusters are abbreviated by capital letters in brackets, following each numbered gBlock D: Deltaproteobacteria. E: Environmental cluster I. F: Firmicutes FACA group sensu lato. N: Nitrospirae. A: Archaeoglobus. Dark blue circle: gBlocks that contain the primer-binding site of DSRp2060F/DSR4R. Green circle: gBlocks with the primer binding site of DSR1728Fmix/DSR4Rmix. Red circle: gBlocks that contain the primer-binding sites of DSR1762Fmix/DSR2107Rmix. Some gBlocks include primer-binding sites for two primer sets. They have been placed in the overlapping areas of the respective primer set circles indicated by teal, purple, and mixed yellow colors. The central white area in the middle of the Venn diagram contains four gBlocks with primer-binding sites for all three primer sets used in this study.

We selected 12 different *dsrB* gene sequences from a recently published *dsrAB* gene sequence database for all qPCR experiments and evaluations in this study based on their phylogenetic diversities and primer binding possibilities [17]. The 12 individual sequences covered most of the current *dsrB* gene sequence diversity, containing representatives among the four phylogenetic superclusters of sulfate-reducing microorganisms, such as *Deltaproteobacteria*, *Firmicutes*, *Nitrospirae*, and the Environmental cluster 1, as well as an archaeal *dsrB* gene from the group *Archaeoglobus* (Fig 1). After the sequence alignment of these 12 *dsrB* genes, a standard fragment of 390 bp was selected and synthesized as double-stranded DNA fragments (gBlocks) by UMGC (S5 Table in S1 File). Each of the selected gBlock sequence contained at least one primer binding site of the three primer sets described above, which can then be classified into three groups according to their primer binding possibilities (based on the in-silico analysis, allowing for up to 1 MM). The three gBlock groups were labeled “common,” “less common,” and “rare,” with primer binding sites for all three, only two, or just one primer set, respectively (Fig 1). The actual amplification performance of each gBlock sequence by different primer sets was further evaluated by qPCR reactions (S6 Table in S1 File). According to our design, nine gBlock sequences contained the primer binding sites for DSR1762Fmix/DSR2107Rmix (Red circle in Fig 1). Eight gBlocks had primer binding sites for DSR1728Fmix/DSR4Rmix (Green circle in Fig 1), and seven gBlocks contained primer binding sites for DSRp2060F/DSR4R (Blue circle in Fig 1). Overall, more gBlock sequences contained primer binding sites of highly degenerate primer sets, since these primers should theoretically have a better sequence coverage (S1 Table in S1 File). The 12 gBlock sequences were mixed to make artificial *dsrB* gene gBTMs, following the assemble strategies below.

#### gBTMs assemble strategies

To assemble *dsrB* gBlock sequences to construct gBTMs showing different evenness and dominance, we mixed all 12 gBlock sequences according to the strategies shown in S7 Table in S1 File. Each gBTM was either dominated by common gBlock sequences (common gBTMs) or rare gBlock sequences (rare gBTMs). The relative abundance of each gBlock sequence in all gBTMs followed a log-normal distribution with the following parameter settings: μ = 1 / σ = 1, μ = 1 / σ = 2, and μ = 1 / σ = 5 (S2 Fig in S1 File). A high σ value indicates higher community unevenness, meaning that only a few gBlock sequences dominated in these gBTMs. In contrast, a low σ value describes gBTMs with a more even relative sequence abundance among the 12 gBlock sequences. In addition, each gBTMs has high and low template concentration conditions.

### Theoretical qPCR quantification performance of each primer set

Before the empirical qPCR tests, we calculated a theoretical template amplification percentage for each gBTMs by different qPCR assays to estimate the effect of target gene sequence evenness and dominance on qPCR testing results and compare the quantification performance among various qPCR assays. This process was done by summing up the relative abundances of all gBlock sequences in the gBTMs that could be amplified by each primer set. We assumed that all gBlock sequences in a gBTMs with more than 1 MM (> 1 MM) to a specific primer set will not be amplified and that all gBlock sequences with 1 or no MM (≤ 1 MM) to a primer set will be amplified at 100% efficiency without any inhibition. While this was a purely theoretical assumption, it allowed us to calculate the theoretical template amplification percentages for all gBTMs under different conditions.

### Empirical qPCR quantification performance of each primer set

The empirical gBTMs amplification percentage of each assembled gBTMs with different target gene sequence evenness, dominance, and concentration conditions was calculated, which is the ratio of qPCR quantification results to total template abundance added to PCR reactions for each primer set. They were then compared against calculated theoretical template amplification percentages to assess the quantification performance of each qPCR assay. Empirical gBTMs amplification percentages were also compared among three qPCR assays at each gBTMs condition for further quantification performance evaluation. All qPCR reactions were performed in triplicate. One-sample and paired t-tests were used to compare the quantified *dsrB* gene copy number of each gBTMs between every two experimental conditions. It was considered significant difference of quantification results between two qPCR assays when the adjusted p-values (according to the Benjamini–Hochberg method) were smaller than 0.05 and when at least a four-fold numerical difference of above qPCR quantification results was observed.

## Results and discussion

Next-generation sequencing technologies continue to contribute to increasing microbial genome sequence information diversity. However, library construction and amplification render sequencing approaches non-quantitative, so that complementary methods such as qPCR and microscopic cell counts are often used to allow for more quantitative analysis of sequence data. The use of qPCR may provide an absolute gene copy numbers representing microbial ribotypes or specific functional gene groups in environmental samples. Furthermore, many practical applications demand the quantification of microorganisms, such as monitoring pathogens in drinking water or wastewater samples. Therefore, understanding the processes linking amplicon reads to species’ cell or gene counts is particularly relevant for a better understanding of ecological processes driving community structure and assembly.

Whereas many qPCR primers designed to quantify 16S rRNA and functional genes are broadly conserved, no primer set is truly universal because of base-pairing exceptions in one or more sequences targeted by conserved primers [21]. The increasing diversity in the microbial sequence database leads to the design of more ambiguous primer sequences to ensure the most comprehensive coverage of target gene sequences [22–25]. As a result, the extent of application of highly degenerate primers on qPCR-based quantification of gene copy numbers in environmental DNA extracts requires systematic experimental evaluation. It is generally believed that degenerate primers are prone to introduce amplification bias due to the primer-template MMs or various primers’ GC content, which limits their applicability [1, 6]. However, it is lack of study on how microbial community characteristics affect the qPCR quantification results using degenerate primers. Thus, we explored two hypotheses as follows: 1) target gene sequence evenness and dominance in environmental samples affect absolute qPCR amplification results; 2) scientific primer choice can help to minize such effect on qPCR results if primer sequences are carefully selected or tuned to template sequence composition.

### qPCR quantification prediction of assembled gBTMs

**Fig 2:**
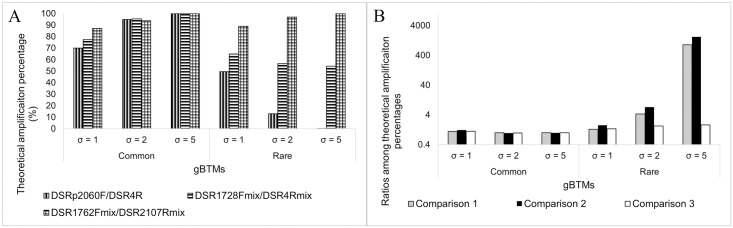
Theoretical amplification performance of all gBTMs by different qPCR assays. (A) Theoretical template amplification percentages of each gBTMs by different qPCR assays. (B) Comparison of theoretical template amplification percentages of each gBTMs among different experimental conditions. Gray (Comparison 1): ratio of theoretical template amplification percentages of each gBTMs by the DSR1728Fmix/DSR4Rmix assay to those by the DSRp2060F/DSR4R assay at each experimental condition. Black (Comparison 2): ratio of theoretical template amplification percentages of each gBTMs by the DSR1762Fmix/DSR2107Rmix to those by the DSRp2060F/DSR4R assay at each experimental condition. White (Comparison 3): ratio of theoretical template amplification percentages of each gBTMs by the DSR1762Fmix/DSR2107Rmix to those by the DSR1728Fmix/DSR4Rmix assay at each experimental condition. The degeneracy of DSRp2060F/DSR4R, DSR1728Fmix/DSR4Rmix assay, and DSR1762Fmix/DSR2107Rmix are 4/1, 77/10, and 98/29, respectively.

Fig 1 gives an overview of the primer binding site occurrences for all 12 gBlock sequences, if allowing for up to one MM. Based on the assumptions made in the method section, a theoretical template amplification percentage for each gBTMs amplified by all primer sets was calculated (Fig 2A and S8 Table in S1 File). We intended to make the theoretical template amplification percentages of all common gBTMs not show a larger than four-fold difference across all evenness conditions (Fig 2B). Similarly, all qPCR assays have comparable theoretical template amplification percentages (less than four-fold changes) for rare gBTMs in the least uneven condition (μ = 1, σ = 1). In contrast, both qPCR assays with highly degenerate primers detected a higher *dsrB* gene copy number than the other one (larger than four-fold changes) for quantification of gBTMs with more considerable unevenness of both μ = 1, σ = 2 and μ = 1, σ = 5 (Fig 2B). Between both highly degenerate qPCR assays, the theoretical template amplification percentages of all gBTMs under each evenness condition showed no significant difference. Such gBTMs design can address our hypothesis about whether and how the target gene sequence evenness and dominance would affect the qPCR quantification results based on empirical quantification and statistical analysis results. In addition, through this experimental setup, we were able to systematically evaluate the performance of highly degenerate primer sets on qPCR quantification compared to a more target-specific primer set.

### Empirical qPCR quantification of assembled gBTMs

**Fig 3:**
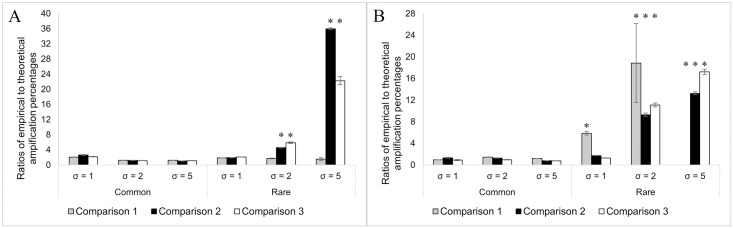
Comparison of theoretical template amplification percentages and empirical qPCR quantification results. (A) The ratio of theoretical template amplification percentages to empirical qPCR quantification results under high template concentration conditions. (B) The ratio of theoretical template amplification percentages to empirical qPCR quantification results under a low template concentration. Gray (Comparison 1): Comparison between theoretical template amplification percentages and empirical qPCR quantification results of the DSRp2060F/DSR4R assay under different conditions. Black (Comparison 2): Comparison between theoretical template amplification percentages and empirical qPCR quantification results of the DSR1728Fmix/DSR4Rmix assay under other conditions. White (Comparison 3): Comparison between theoretical template amplification percentages and empirical qPCR quantification results of the DSR1762Fmix/DSR2107Rmix assay under different conditions. The degeneracy of DSRp2060F/DSR4R, DSR1728Fmix/DSR4Rmix assay, and DSR1762Fmix/DSR2107Rmix are 4/1, 77/10, and 98/29, respectively.

Fig 3 compares empirically determined quantification results and theoretical template amplification percentages. In general, no matter what template concentration conditions were used, the empirical quantification results of common gBTMs were agreeable to corresponding theoretical template amplification percentages very well (all fold changes were less than four, as shown in Fig 3A and 3B). Under the high template concentration condition, the qPCR assay of DSRp2060F/DSR4R also showed the expected quantification results for all rare gBTMs based on their theoretical template amplification percentages (Fig 3A). However, significantly lower quantification results of rare gBTMs were observed from qPCR assays of DSR1728Fmix/DSR4Rmix and DSR1762Fmix/DSR2107Rmix when the target gene sequence unevenness was μ = 1, σ = 2 and μ = 1, σ = 5 (Fig 3A). This suggested that highly degenerate primer sets adversely affected their quantification performance. Such effects may be primarily caused by the complexity and low overall amplification efficiency of the whole qPCR assay, which is supported by the qPCR testing results using individual gBlocks as reaction templates (S6 Table in S1 File). Specifically, the dominant sequences in rare gBTMs, including gBlock 3, 6, and 7, were poorly amplified by both DSR1728Fmix/DSR4Rmix and DSR1762Fmix/DSR2107Rmix, showing significantly lower quantification results than the template concentration (only ranging from 1.66% to 8.55% of template abundance added to the qPCR reactions listed in S6 Table in S1 File). Furthermore, S6 Table in S1 File suggested that the qPCR amplification performance was not always reflected by the number of MM between primers and their corresponding binding regions. For example, the primer binding regions of gBlock 3 had <1 MM for the primer set of DSR1762Fmix/DSR2107R, but the amplification efficiency was only 6.71%. In contrast, the qPCR assays of DSRp2060F/DSR4R and DSR1728Fmix/DSR2107Rmix showed comparable amplification efficiency of gBlock 1, but only former primer set had <1 MM against the primer binding regions of gBlock 1. Such phenomenon further complexed the PCR amplification performance in each unique PCR assay. These findings are consistent with the conclusions drawn in previous publications [1, 6]. Substantial variations of amplification efficiencies were observed in qPCR assays with degenerate primers, and quantification error could be up to orders of magnitude sometimes. In short, either target gene sequence evenness or dominance alone can fully explain the qPCR quantification behaviors of all qPCR assays tested in this study. The qPCR quantification performance is determined by the comprehensive effect of both the target gene sequence evenness and dominance. All qPCR assays have even lower quantification performance when the template concentration is low. Only empirical qPCR quantification results from qPCR assays of DSR1728Fmix/DSR4Rmix and DSR1762Fmix/DSR2107Rmix in the least uneven condition (μ = 1, σ = 1) were close to the estimated ones (Fig 3B). The study by Brankatschk et al. reported that high template concentration (>10^6^ copies/μL) may inhibit the qPCR amplification, and low template concentration could also cause adverse effects on the qPCR quantification, especially for the poorly amplifiable sequences [1]. Our findings partially agree with their conclusions since we didn’t see more significant inhibition of high template concentrations than low ones on qPCR quantification. However, the low template concentration condition was confirmed to be more challenging for accurate qPCR quantification in our study.

**Fig 4:**
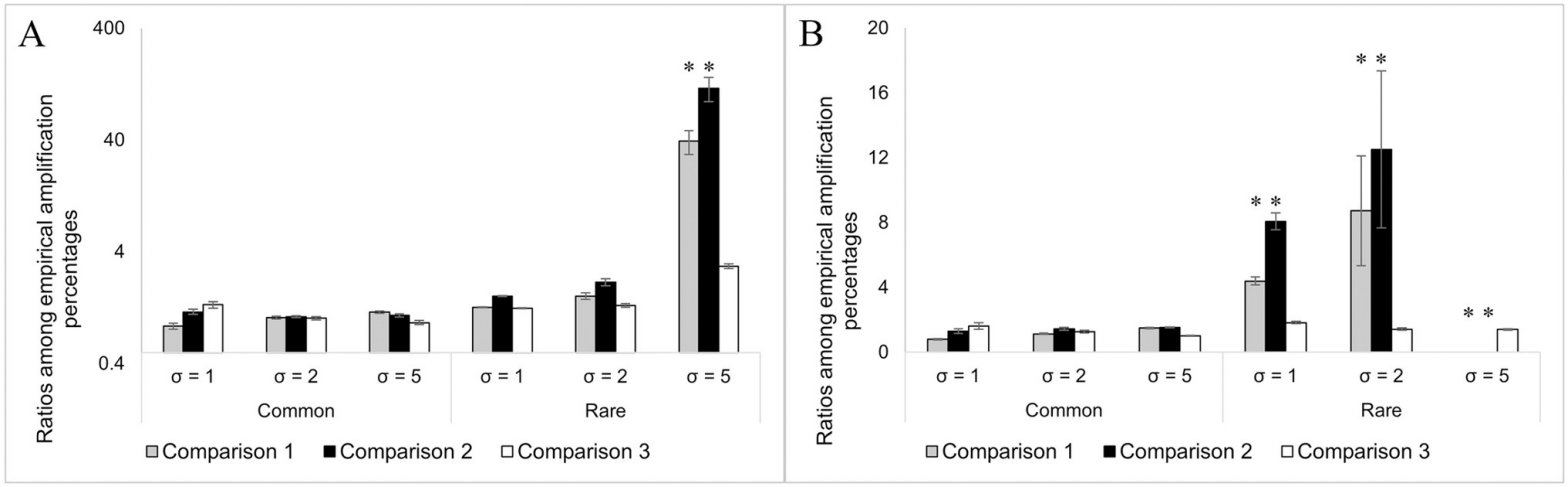
Pairwise comparison of empirical qPCR results between different qPCR assays under high (A) and low (B) template concentration conditions. Gray (Comparison 1): Comparison of empirical qPCR results between the DSR1728Fmix/DSR4Rmix assay and the DSRp2060F/DSR4R assay. Black (Comparison 2): Comparison of empirical qPCR results between the DSR1762Fmix/DSR2107Rmix assay and the DSRp2060F/DSR4R assay. White (Comparison 3): Comparison of empirical qPCR results between the DSR1762Fmix/DSR2107Rmix assay and the DSR1728Fmix/DSR4Rmix assay. The degeneracy of DSRp2060F/DSR4R, DSR1728Fmix/DSR4Rmix assay, and DSR1762Fmix/DSR2107Rmix are 4/1, 77/10, and 98/29, respectively.

Fig 4 shows the empirical quantification results comparison of all gBTMs among the three qPCR assays. Overall, qPCR quantification performance on common gBTMs is comparable among all three qPCR assays, as estimated (Fig 4A and 4B). For rare gBTMs under high template concentration conditions, other than the two evenness conditions suggested by Fig 3A, both highly degenerate primer sets only worked better than primer set DSRp2060F/DSR4R when the evenness condition was μ = 1, σ = 5 (Fig 4A). This is mainly due to the PCR biases caused by the highly degenerate primer sets of DSR1728Fmix/DSR4Rmix and DSR1762Fmix/DSR2107Rmix, as described above. The significant PCR biases in qPCR assays of both highly degenerate primer sets essentially reduce the difference in quantification results between them and the qPCR assay of DSRp2060F/DSR4R. Under the low template concentration condition, both primer sets DSR1728Fmix/DSR4Rmix, and DSR1762Fmix/DSR2107Rmix worked better than primer set DSRp2060F/DSR4R on quantification of rare gBTMs across all evenness conditions (Fig 4B). However, all three qPCR assays showed overall poor quantification performance. This indicates that the low template concentration could exaggerate the difference in quantification results between the qPCR assay of DSRp2060F/DSR4R and the other two, and highly degenerate primer sets would be more resistant to the adverse effect of low template concentration on qPCR quantification. In summary, highly degenerate primer sets have either equivalent or better performance on qPCR quantification of *dsrB* gene gBTMs made in this study, which mimics the different situations used to quantify *dsrB* gene abundance in culture-enriched or typical environmental samples (e.g., activated sludge) using qPCR methods. A comparison of empirical quantification results also proved and explained the effect of target gene sequence evenness and dominance on qPCR quantification.

Based on our own findings we suggest that it is not necessary a suboptimal option to use highly degenerate primer sets for qPCR quantification of target genes, especially when no prior knowledge about the gene sequence information of tested samples is available. Considering that access to large amounts of high-throughput sequencing data has become more and more affordable, it may be worth making metagenomic sequencing data availability a prerequisite for the application of qPCR in environmental studies since prior knowledge of target gene sequence composition and structure will provide a guide for qPCR primer selection and reaction condition optimization, resulting in more accurate qPCR quantification.

## Conclusions

In this study, we investigated the effect of target gene sequence evenness and dominance on qPCR quantification of defined *dsrB* gene template mixtures using three primer sets with different levels of degeneracy. We further compared the qPCR quantification performance among these qPCR assays under different template mixture and concentration conditions. Our study showed the following:

1. The effects of target gene sequence evenness or dominance on qPCR quantification results depend on each other.
2. PCR-biases may cause significantly poor amplification of target gene sequences by highly degenerate primer sets when unevenness increases, especially under the low template concentration condition, but they still work as well as or even better than more target-specific primer sets on qPCR quantification of all gBTMs in this study.
3. Knowing how well primers amplify specific target sequences in a diverse target gene sequence mixture will always be advantageous. This is generally not possible in environmental DNA studies unless prior knowledge of metagenomic sequencing data exists. Given that metagenomic sequencing data on any environmental sample can nowadays be easily accessed or generated, the availability of sequence data will allow for careful evaluation of primer or target complementarity and rational reaction condition optimization to increase the accuracy and precision of qPCR results in environmental microbiology and microbial ecology.

## Supporting information

S1 File (DOCX)
