# Supplementary material for: Effect of target gene sequence evenness and dominance on real-time PCR quantification of artificial sulfate-reducing microbial communities
Source: PLoS One. 2024 Mar 7;19(3):e0299930. doi: 10.1371/journal.pone.0299930 (PMC10919606; doi:10.1371/journal.pone.0299930)
Supplement: S1 File — (DOCX) [file pone.0299930.s001.docx]

**Supporting Information**

**Effect of target gene sequence evenness and dominance on real-time PCR quantification of artificial sulfate-reducing microbial communities**

Zhe Du^1,2*^ and Sebastian F. Behrens^2,3^

^1^Center for Environmental Health Risk Assessment and Research, Chinese Research Academy of Environmental Sciences, Beijing 100012, China

^2^The BioTechnology Institute, University of Minnesota Twin Cities, St. Paul, Minnesota, United States of America

^3^Department of Civil, Environmental, and Geo-Engineering, University of Minnesota Twin Cities, Minneapolis, Minnesota, United States of America

*Corresponding author:

E-mail: [du.zhe@craes.org.cn](mailto:du.zhe@craes.org.cn); [maxduzhe@hotmail.com](mailto:maxduzhe@hotmail.com)

S1 Table. Three primer sets used in this study for qPCR assays targeting the *dsrB* gene.

| Name | Sequence (5’-3’) | Deg^a^ | GC % | Tm | Position^b^ | PPL^c^ | All dataset *dsrAB*^d^ | | Reference |
| --- | --- | --- | --- | --- | --- | --- | --- | --- | --- |
|  |  |  |  | ($℃$) |  |  | 0 MM | 1 wMM |  |
| DSRp2060F | CAACATCGTYCAYACCCAGGG | 4 | 57.1 | 58.1 | 1752-1772 | 378 | 7.91 | 18.61 | Geets *et al* ., 2006[1] |
| DSR4R | GTGTAGCAGTTACCGCA | 1 | 52.9 | 52.0 | 2113-2129 |  | 7.26 | 10.67 | Wagner *et al* ., 1998[2] |
|  |  |  |  |  |  |  |  |  |  |
| DSR1728Fmix |  | 77 | 59.4 |  |  | 368 | 36.84 | 39.64 | Steger *et al* ., 2011[3] |
| DSR1728F1 | CAYACCCAGGGNTGG | 8 | 66.7 | 52.2 | 1762-1776 |  | 24.11 | 35.37 | Steger *et al* ., 2011 |
| DSR1728F2 | CAYACBCAAGGNTGG | 24 | 57.8 | 48.5 | 1762-1776 |  | 3.03 | 35.43 | Steger *et al* ., 2011 |
| DSR1728F3 | CATACDCAGGGHTGG | 9 | 57.8 | 47.8 | 1762-1776 |  | 2.43 | 16.19 | Steger *et al* ., 2011 |
| DSR1728F4 | CACACDCAGGGNTGG | 12 | 65.6 | 52.4 | 1762-1776 |  | 6.51 | 27.06 | Steger *et al* ., 2011 |
| DSR1728F5 | CATACHCAGGGNTAY | 24 | 48.9 | 43.2 | 1762-1776 |  | 0.77 | 2.57 | Steger *et al* ., 2011 |
|  |  |  |  |  |  |  |  |  |  |
| DSR4Rmix |  | 10 | 48.8 |  |  |  | 3.87 | 11.96 |  |
| DSR4Ra | GTGTAACAGTTTCCACA | 1 | 41.2 | 46.4 | 2113-2129 |  | 0.04 | 0.31 | Loy *et al* ., 2004[4] |
| DSR4Rb | GTGTAACAGTTACCGCA | 1 | 47.1 | 49.2 | 2113-2129 |  | 0.40 | 8.03 | Loy *et al* ., 2004 |
| DSR4Rc | GTGTAGCAGTTKCCGCA | 2 | 55.9 | 54.3 | 2113-2129 |  | 2.37 | 10.94 | Loy *et al* ., 2004 |
| DSR4Rd | GTGTAGCAGTTACCACA | 1 | 41.2 | 48.6 | 2113-2129 |  | 0.18 | 7.76 | Zverlov *et al* ., 2005[5] |
| DSR4Re | GTGTAACAGTTACCACA | 1 | 41.2 | 45.8 | 2113-2129 |  | 0.08 | 0.79 | Zverlov *et al* ., 2005 |
| DSR4Rf | GTATAGCARTTGCCGCA | 2 | 50.0 | 51.3 | 2113-2129 |  | 0.13 | 1.46 | Pester *et al* ., 2010[6] |
| DSR4Rg | GTGAAGCAGTTGCCGCA | 1 | 58.8 | 56.6 | 2113-2129 |  | 0.68 | 1.88 | Pester *et al* ., 2010 |
|  |  |  |  |  |  |  |  |  |  |
| DSR1762Fmix |  | 98 | 58.4 |  | 1762-1776 | 362 | 38.64 | 39.99 |  |
| DSR1762F1 | CAYACCCAGGGNTGG | 8 | 66.7 | 52.2 | 1762-1776 |  | 24.11 | 35.37 | Steger *et al* ., 2011[3] |
| DSR1762F2 | CAYACBCAAGGNTGG | 24 | 57.8 | 48.5 | 1762-1776 |  | 3.03 | 35.43 | Steger *et al* ., 2011 |
| DSR1762F3 | CATACDCAGGGHTGG | 9 | 57.8 | 47.8 | 1762-1776 |  | 2.43 | 16.19 | Steger *et al* ., 2011 |
| DSR1762F4 | CACACDCAGGGNTGG | 12 | 65.6 | 52.4 | 1762-1776 |  | 6.51 | 27.06 | Steger *et al* ., 2011 |
| DSR1762F5 | CATACHCAGGGNTAY | 24 | 48.9 | 43.2 | 1762-1776 |  | 0.77 | 2.57 | Steger *et al* ., 2011 |
| DSR1762F6 | CACACSCAGGGKTAY | 8 | 60.0 | 50.1 | 1762-1776 |  | 0.61 | 1.85 | Pelikan *et al* ., 2016[7] |
| DSR1762F7 | CACACBCAGGGMTAC | 6 | 61.1 | 49.4 | 1762-1776 |  | 0.53 | 1.60 | Pelikan *et al* ., 2016 |
| DSR1762F8 | CACACHCAGGGCTAT | 3 | 55.6 | 48.5 | 1762-1776 |  | 0.40 | 1.35 | Pelikan *et al* ., 2016 |
| DSR1762F9 | CACACCCAGGGWTTC | 2 | 60.0 | 49.3 | 1762-1776 |  | 0.12 | 0.68 | Pelikan *et al* ., 2016 |
| DSR1762F10 | CAYACACAAGGATGG | 2 | 50.0 | 44.5 | 1762-1776 |  | 0.13 | 0.84 | Pelikan *et al* ., 2016 |
|  |  |  |  |  |  |  |  |  |  |
| DSR2107Rmix |  | 29 | 46.8 |  | 2107-2123 |  | 16.27 | 17.01 |  |
| DSR2107R1 | CAGTTDCCRCARTACAT | 12 | 43.1 | 47.4 | 2107-2123 |  | 7.36 | 16.75 | Lever *et al* ., 2013[8] |
| DSR2107R2 | CAGTTACCRCAGAACAT | 2 | 44.1 | 47.6 | 2107-2123 |  | 6.55 | 13.70 | Lever *et al* ., 2013 |
| DSR2107R3 | CAGTTGSCGCAGAACAT | 2 | 52.9 | 53.3 | 2107-2123 |  | 1.40 | 9.99 | Lever *et al* ., 2013 |
| DSR2107R4 | CAGTTYCCGCAGAACAT | 2 | 50 | 51.2 | 2107-2123 |  | 0.84 | 9.51 | Pelikan *et al* ., 2016[7] |
| DSR2107R5 | CAGTTKCCACAGAACAT | 2 | 44.1 | 48.2 | 2107-2123 |  | 0.12 | 2.77 | Lever *et al* ., 2013[8] |

^a^Degeneracy is given as the number of oligonucleotides that comprise the primer.

^b^Position is relative to *Desulfovibrio vulgaris* Hildenborough *dsrAB* gene.

^c^PCR product length.

^d^The curated and annotated *dsrAB* sequences database (n = 7695) published by Müller *et al*., 2015 is used to calculate the in silico sequence coverage (%) of each primer or primer mix.

S2 Table. Reaction setup of different qPCR assays.

|  |  |  | DSRp2060F/DSR4R | DSR1728Fmix/DSR4Rmix | DSR1762Fmix/DSR2107Rmix |
| --- | --- | --- | --- | --- | --- |
| qPCR reaction setup  (concentration / rxn) | SsoFast^TM^ EvaGreen^®^ Supermix | | 1x | 1x | 1x |
|  | Forward primer | | 400 nM | 400 nM^a^ | 400 nM^c^ |
|  | Reverse primer | | 300 nM | 300 nM^b^ | 400 nM^d^ |
|  | DNA template | | - | - | - |
|  | Bovine Serum Albumin | | 0.5 µg/µl | 0.5 µg/µl | 0.5 µg/µl |
|  | H_2_O | | - | - | - |
|  |  |  |  |  |  |
| qPCR thermocycler protocol  (Temperature / Time) | Initial denaturation | | 95 $℃$ / 5 min | 95 $℃$ / 5 min | 95 $℃$ / 5 min |
|  | Denaturation | | 95 $℃$ / 30 s | 95 $℃$ / 30 s | 95 $℃$ / 30 s |
|  | Annealing | | 58 $℃$ / 30 s | 55 $℃$ / 30 s | 55 $℃$ / 30 s |
|  | Elongation | | 72 $℃$ / 45 s | 72 $℃$ / 45 s | 72 $℃$ / 45 s |

^a^Each forward primer of DSR1728Fmix has the final concentration of 400 nM in qPCR reaction mixtures.

^b^Each reverse primer of DSR4Rmix has the final concentration of 300 nM in qPCR reaction mixtures.

^c^Each forward primer of DSR1762Fmix has the final concentration of 400 nM in qPCR reaction mixtures.

^d^Each reverse primer of DSR2107Rmix has the final concentration of 400 nM in qPCR reaction mixtures.

S3 Table. Primer concentration optimization of each primer set

| Forward/Reverse Primer Concentration (nM) |  | Average Cq Values^a^ | | |
| --- | --- | --- | --- | --- |
|  |  | DSRp2060F/DSR4R | DSR1728Fmix/DSR4Rmix^b^ | DSR1762Fmix/DSR2107Rmix^c^ |
| 500/500 | | 10.89 | 11.23 | 11.94 |
| 400/400 | | 10.97 | 11.02 | 11.96 |
| 400/300 | | 10.40 | 10.68 | 12.19 |
| 300/400 | | 10.66 | 11.17 | 12.49 |
| 300/300 | | 10.82 | 12.73 | 12.96 |

^a^Average Cq values were from qPCR reactions of each primer set using a 500 bp synthesized fragment of the *dsrB* gene from *Desulfovibrio vulgaris* Hildenborough (0.01 ng/µL) as the template under different primer concentration condition. qPCR experiments were performed in triplicate.

^b^Each primer sequence of DSR1728Fmix/DSR4Rmix has the final concentration shown in the first column.

^c^Each primer sequence of DSR1762Fmix/DSR2107Rmix has the final concentration shown in the first column.

S4 Table. Annealing temperature optimization of each primer set

| Annealing Temperature (°C) |  | Average Cq Values^a^ | | |
| --- | --- | --- | --- | --- |
|  |  | DSRp2060F/DSR4R | DSR1728Fmix/DSR4Rmix | DSR1762Fmix/DSR2107Rmix |
| 52.0 | | 10.48 | 12.48 | 12.68 |
| 53.5 | | 10.27 | 11.65 | 11.91 |
| 55.0 | | 10.20 | 11.08 | 10.93 |
| 56.5 | | 10.32 | 11.38 | 11.29 |
| 58.0 | | 10.04 | 14.81 | 11.32 |
| 59.5 | | 10.13 | 17.45 | 12.51 |
| 62.0 | | 10.39 | 19.23 | 12.73 |

^a^Average Cq values were from qPCR reactions of each primer set using a 500 bp synthesized fragment of the *dsrB* gene from *Desulfovibrio vulgaris* Hildenborough (0.01 ng/µL) as the template under each annealing temperature condition. qPCR experiments were performed in triplicate.

| DSRp2060F/DSR4R |
| --- |
|  |
| DSR1728Fmix/DSR4Rmix |
|  |
| DSR1762Fmix/DSR2107Rmix |
|  |

S1 Fig. Standard curves of each qPCR assay.

S5 Table. Selected *dsrB* gBlock sequences

| gBlock ID | Sequence ID^a^ | Description | Sequences |
| --- | --- | --- | --- |
| 1 | UncSu520 | Continental margin sediment clone | CGGCATCGGCAACTCGATCTCCAACATCGTCCACACCCAGGGCTGGGTGCACTGTCACTCGGCGGCCACCGACGCCTCGGGGGTCGTGAAATCCATCATGGACGAGCTGTACCCCCACTTCGTGGACATGAAGCTCCCGGGGAAGCTCCGCATCGCCTTCGCTTGCTGCCTCAACATGTGCGGCGCCGTGCACTGCTCCGACATCGCGGTCCTCGGCGTGCACCGCGGCCCTCCGAAGATCAACCACGACGAGCTGCCCAAGCTCTGCGAGGTGCCGCACCCTGTGTCGTCGTGTCCCACCGGCGCCATCCGGCCCGCTACGGTCAACGACAAGCCCTCGGTCGAGATCCAGGACGAGCAGTGCATATACTGCGGTAAATGCTACACCAT |
| 2 | UncSu450 | Continental margin sediment clone | CGGCAGCAAGCTCTCCAACATCGTCCACACTCAGGGCTGGGTCCACTGCCACTCAGCGGCGTCGGACGCCTCGGGCGTGGTCAAATCGGTGATGGACGAGCTCTTCCCATACTTCTCCGGCGAGAAGGACCTGCCGGCGAAGACGAGGATCGCCTACGCCTGCTGTCTCAACATGTGTGGTGCGGTCCACTGCTCCGACATCGCGATCCTCGGGGTTCACACCCGGGCGCCGGTCATCAACCACGACGATCTGCCCAAGATGTGCGAGATCCCGACCCTGGTCGCCTCGTGTCCCACAGGCGCCATCCGGCCGGCGACGGTCGACGGAGTGCAATCGGTGGAAATCGTCGATGAGCAGTGCGTGTACTGCGGAAAATGCTACACCAGTTC |
| 3 | FJ040818 | Soil metagenome | GGGCGGCCTGGGCAACTCGATCTCCAACATCGTGCACACGCAGGGTTGGGTGCATTGCCACTCGGCGGCCACGGACGCTTCCGGCGTCGTCAAGTGCGTGATGGACGATCTGCACGAGTACTTCACGGAGATGAAGCTGCCGGGCAAGCTGCGGATCGCGCTGGCCTGCTGCCTGAACATGTGCGGTGCCGTGCATTGTTCGGACATTGCCATCCTGGGCGTCCACCGGCGCCCGCCGAAGGTCCAGCACGACAACCTGAACAAGCTCTGCGAGATCCCCAACGTGGTGGCCTCCTGCCCCACGGCCGCGATCCGGCCCACGACCGTCGACGGCAAGCCTTCGGTCGAGGTCCTGGAGGAGCGGTGCATGTTCTGCGCCAACTGCTTCTC |
| 4 | JX068858 | uncultured bacterium | CGATTCAACATCGTTCACACCCAGGGATGGATCCACTGCCATACCCCGGCAACGGATGCTTCCGGCCCGGTCAAGGCCACCATGGACGTGCTCTTTGACGACTTCAAAGATCACCGCCTGCCGGCCCAGCTGCGCGTCTCTTTGGCCTGCTGCCTGAACATGTGCGGTGCCGTGCACTGCTCCGACATCGCCATTCTCGGCTACCATAGAAAACCGCCGCTGATCGATAACGAATATCTGGACAAAATGTGTGAAATTCCCTTGGCCATCGCCTCGTGCCCAACCGCGGCCATCAAACCGACCAAGGTGAAAGTGGGTGATAAGGAAGTCAAGTCCGTGGCCGTGAACAACGATCGCTGCATGGCTTGCGGTAACTGCTACACAATCACT |
| 5 | JF906916 | uncultured bacterium | GGGCCCAACATCGTCCACACCCAGGGTTTCATCCACTGTCATACCCCTGCAACCGATGCGTCCAGTATGGTAAAGGCCACCATGGACGTGCTTTTTGATGATTTTAAAAAGATGCGTTTGCCGGCGCAGATGCGCGTCTCTTTGGCCTGCTGCTTGAATATGTGCGGCGCGGTGCACTGCTCGGATATTGCTATCCTGGGCTACCATCGCAAGCCGCCCTTGATGGACCATGAATACCTGGACAAATTGTGCGAAATTCCGCTGGCCATCGCGTCCTGTCCCACGGCAGCGCTCAAGCCGGGGAAAGTGGAACTGGAAGACGGCACCTCGGTTAAAAGCATTATCGTCAACGAGCCCCGCTGTATGTTCTGCGGTAACTGCTACACAGTG |
| 6 | CanDesu2 | *Desulfococcus oleovorans* | CACCAACATCATTCACACGCAAGGGTGGATTCACTGTCACACCCCGGCAACGGATGCTTCCGGCCCTGTAAAGGCGACCATGGACGCCCTGTTTGACCACTTCCAGAGCATGGACCTGCCGGCCCAGGTGCGGGTCTCCCTGGCCTGCTGCCTGAACATGTGCGGCGCGGTGCACTGCTCGGATATCGCCATTCTGGGCTACCACAGAAAACCGCCCATGATCGAGGACGAGTACCTTGACAAGATGTGCGAAATTCCGCTGGCGGTGGCGGCCTGCCCCACGGCGGCCATCAAGCCCGTCAAAAAGACCCTTGAGGACGGCACCGAAGTCAAGACCGTGGCCGTGAACAACGAGCGGTGCATGTACTGCGGCAACTGCTACACCATGTG |
| 7 | FJ812101 | uncultured bacterium | ATGATTGGTGGTATTGGCTCAAGAGTAAGCAATATAGTTCATACACAGGGATGGATTCACTGCCATTCAGCAGCTGTTGATGCATCTGCTCTCGTTAAGGCTATTATGGATGAGTTTGCTGATTACTTTACAACAAAAGAGACCCCAAATAAAGTGAGATTTGCTGTTGCCTGCTGCACAAATATGTGCGGTGCAGTTCATTGCTCTGATATTGCTTTCGTTGGAATTCACAGAAAGCTTCCAAGAATTGACCATGACAACTTCAAAAACCTCTGTGAGCTTCCCACAACGATGGCTTCATGCCCAACTGCAGCAATTACACCTGATCCTGCAAAGAAATCTATAAAGATTAATGCTGAGAAATGCATGTACTGCGGTAACTGTTACACA |
| 8 | UncS1648 | Polluted aquifer clone | GTCACCAACATCGTCCACACCCAGGGCTGGATCCACTGCCATACGCCGGCTACGGACGCATCAGGTCCGGTAAAAGCCACCATGGATGTGCTTTTCGATGATTTCCAGAATATGCGGCTTCCTGCCCAGCTTCGCGTATCTTTGGCATGCTGTTTGAACATGTGCGGCGCTGTCCACTGTTCGGACATCGCCATTCTGGGGTATCACCGCAAACCACCCATGCTGGATCATGAATACCTGGACAAGATGTGTGAAATTCCCCTGGCCATTGCCGCCTGCCCCACGGCAGCCATCAAGCCGTCAAAGATGGAACTTGCCAATGGAAATAAGGTGAACAGTGTTTCGGTTAATAATGAAAGATGTATGTACTGCGGTAACTGCTACACAAGA |
| 9 | UncS1652 | Polluted aquifer clone | CATCGGCAACAGCATCTCCAACATCGTCCACACCCAGGGCTGGGTGCACTGCCACTCGGCGGCCTCCGACGCCTCGGGTGTGGTCAAGTGCCTGATGGACGAGCTCTACCCGCATTTCGCCGACCGGAAGCTGCCGGCGAAGCTGCGCGTTGCCTTCGCCTGCTGCCTCAACATGTGCGGCGCCGTGCACTGCTCGGACATCGCGGTCCTGGGCGTCCACACCCGTCCACCGAAGGTCAACCACGAGGAGCTGCCGCGGATGTGCGAAGTCCCGACCGCGGTCGCCTCCTGTCCCACCGGTGCCATCCGCCCGGCCACGGTCAGTGGCAAGGCGTCGGTCGAGGTGATCGACGAGCAGTGCATGTACTGCGGTAACTGCTACACAGACCT |
| 10 | DscHydr2 | *Desulfotomaculum hydrothermale* | TGGCGGTACCGGCAACTCCATTACCAACATCGTACACACCCAGGGTTGGGTACACTGCCATACCCCGGCTACCGATGCTTCCGGTATTGTGAAAGCCGTTATGGATGAGCTGTTTGAGTACTTCACCACCATGAAGCTGCCTGCCAAGCTGAGAATCGCTCTGGCTTGCTGCTTGAACATGTGCGGTGCTGTACACTGCTCCGACATCGCCATTCTGGGTATTCACAGAACCGTACCCAAAATCGATCACGAAAACCTGCATAAGCTGTGCGAGATTCCCACTCTGACCGCCAGCTGCCCCACTGCAGCCATTCGTCCTAACCCCAAACTGAAGTCTATCGAAATTAAGGCCGAACGCTGCATGTACTGCGGTAACTGCTACACCATGTG |
| 11 | JF906943 | uncultured bacterium | GGGCACGGGGGGCCCAACATCGTCCACACCCAGGGATGGGTGCACTGCCACAGTGCATGTACAGATGCTTCAGGCATAGTGAAGGCTGTCATGGATGAACTCTATGACTACTTCACAACAAAAGAACTGCCTAACAAAGTGAGACTTGCAGTTGCGTGCTGTGTAAACATGTGCGGTGCTGTTCACTATTCTGACATCGCGGTGGTTGCCGTGCACAGAAAAGTACCGACAGTAGACGAAGAAAATGTAGGGAAAATGTGTGAAATTCCGACAACTGTTGCTTCACGACCGACTTCGGCTATCAGACCGAATCCAAAGACAAAATCAATTACTATTAATGATGAAAAGTGCATGTACTGCGGTAACTGCTACACACCACTACGCACTGGA |
| 12 | ArgVene3 | *Archaeoglobus veneficus* | ACGTGGGACACCCCAAGTGGTAAGTATGCTCTCTCAAACATCATCCACACCCAGGGCTGGATACACTGCCACACGCCCGCTATCGACGCTTCGGGTATTGTGAAGGCTATCATGGACGAACTCTATGAGTACTTTGTTGACATGAAACTGCCGGCAATGTGCAGAATCAGCCTTGCATGCTGTGCTAACATGTGTGGAGCAGTTCACGCTTCGGACATATCCATTGTCGGTATCCACAGGACTCCTCCGCTCGTTGATGACGACGCAGTGAAGAGGATGTGTGAGCTGCCAACTACTGCTGCATCATGTACAACGGCGCAATTAGATCAAGGCCAAGAGAAGACTGTCGAGGTTGACGGCGAGAAGTGCATGTACTGCGGCAACTGCTAC |

^a^Sequence ID is from the *dsrAB* sequences database (n = 7695) published by Müller *et al*., 2015

S6 Table. Quantification performance of each gBlock sequence by different primer sets

|  | gBlock amplification percentage (%) | | |
| --- | --- | --- | --- |
|  | DSRp2060F/  DSR4R | DSR1728Fmix/  DSR4Rmix | DSR1762Fmix/  DSR2107Rmix |
| gBlock 1 | 69.67 | 76.94 | - |
| gBlock 2 | 2.56 | 20.99 | - |
| gBlock 3 | - | 1.66 | 6.71 |
| gBlock 4 | 99.16 | 42.74 | - |
| gBlock 5 | 64.54 | - | 577.02 |
| gBlock 6 | - | 3.54 | 5.39 |
| gBlock 7 | - | 8.55 | 4.70 |
| gBlock 8 | 95.32 | 97.45 | 108.14 |
| gBlock 9 | 73.52 | 5.34 | 2.68 |
| gBlock 10 | 60.27 | 49.58 | 36.33 |
| gBlock 11 | 41.59 | 11.11 | 4.40 |
| gBlock 12 | 7.27 | 91.90 | 182.08 |

Note:

gBlock amplification percentage = empirically determined gBlock sequence abundance / template abundance added to qPCR reactions

S7 Table. *dsrB* gene gBTMs assembling strategies.

| Rank^a^ | gBlocks to construct gBTMs^b^ | | Relative abundance (%) | | |
| --- | --- | --- | --- | --- | --- |
|  | Rare gBTMs | Common gBTMs | µ = 1 / σ = 1 | µ = 1 / σ = 2 | µ = 1 / σ = 5 |
| 1 | 3 | 8 | 14.95 | 32.58 | 45.52 |
| 2 | 7 | 10 | 12.03 | 27.17 | 40.30 |
| 3 | 6 | 9 | 11.32 | 17.01 | 14.06 |
| 4 | 12 | 11 | 10.86 | 9.60 | 6.01 * 10^-2^ |
| 5 | 8 | 4 | 10.53 | 5.62 | 1.47 * 10^-2^ |
| 6 | 10 | 5 | 9.10 | 2.54 | 1.45 * 10^-2^ |
| 7 | 4 | 6 | 8.70 | 2.43 | 1.32 * 10^-2^ |
| 8 | 5 | 7 | 8.14 | 0.99 | 1.30 * 10^-2^ |
| 9 | 11 | 3 | 6.11 | 0.78 | 4.44 * 10^-4^ |
| 10 | 9 | 12 | 5.97 | 0.69 | 3.55 * 10^-5^ |
| 11 | 2 | 1 | 1.26 | 0.48 | 7.45 * 10^-6^ |
| 12 | 1 | 2 | 1.03 | 0.10 | 1.10 * 10^-6^ |

^a^The rank number 1 to 12 indicate the relative abundance of each corresponding gBlock in columns “Rare gBTMs” and “Common gBTMs” from the highest to lowest.

^b^The numbers in the columns “Common gBTMs” and “Rare gBTMs” are the gBlock ID numbers to show gBlocks to construct rare and common gBTMs, respectively.

S2 Fig. gBlock relative abundance distribution in different gBTMs. Diamonds: gBTMs with µ = 1, σ = 1. Squares: gBTMs with µ = 1, σ = 2. Triangles: gBTMs with µ = 1, σ = 5.

S8 Table. Theoretical template amplification percentage of each gBTMs by different qPCR assays.

| qPCR assays | Theoretical template amplification percentage (%) | | | | | |
| --- | --- | --- | --- | --- | --- | --- |
|  | Common | | | Rare | | |
|  | µ = 1, σ = 1 | µ = 1, σ = 2 | µ = 1, σ = 5 | µ = 1, σ = 1 | µ = 1, σ = 2 | µ = 1, σ = 5 |
| DSRp2060F/DSR4R | 70.05 | 95.00 | 99.97 | 49.58 | 13.15 | 0.06 |
| DSR1728Fmix/DSR4Rmix | 77.56 | 95.50 | 99.98 | 65.02 | 56.72 | 54.40 |
| DSR1762Fmix/DSR2107Rmix | 87.18 | 93.79 | 99.98 | 89.01 | 96.98 | 99.98 |

**Reference**

1. Geets J, Borrernans B, Diels L, Springael D, Vangronsveld J, van der Lelie D, et al. DsrB gene-based DGGE for community and diversity surveys of sulfate-reducing bacteria. J Microbiol Meth. 2006;66(2):194-205. doi: 10.1016/j.mimet.2005.11.002. PubMed PMID: WOS:000238963000002.

2. Wagner M, Roger AJ, Flax JL, Brusseau GA, Stahl DA. Phylogeny of dissimilatory sulfite reductases supports an early origin of sulfate respiration. J Bacteriol. 1998;180(11):2975-82. doi: Doi 10.1128/Jb.180.11.2975-2982.1998. PubMed PMID: WOS:000074002100027.

3. Steger D, Wentrup C, Braunegger C, Deevong P, Hofer M, Richter A, et al. Microorganisms with Novel Dissimilatory (Bi)Sulfite Reductase Genes Are Widespread and Part of the Core Microbiota in Low-Sulfate Peatlands. Applied and Environmental Microbiology. 2011;77(4):1231-42. doi: 10.1128/Aem.01352-10. PubMed PMID: WOS:000287078100010.

4. Loy A, Kusel K, Lehner A, Drake HL, Wagner M. Microarray and functional gene analyses of sulfate-reducing prokaryotes in low-sulfate, acidic fens reveal cooccurrence of recognized genera and novel lineages. Applied and Environmental Microbiology. 2004;70(12):6998-7009. doi: 10.1128/Aem.70.12.6998-7009.2004. PubMed PMID: WOS:000225719300008.

5. Zverlov V, Klein M, Lucker S, Friedrich MW, Kellermann J, Stahl DA, et al. Lateral gene transfer of dissimilatory (bi)sulfite reductase revisited. J Bacteriol. 2005;187(6):2203-8. doi: 10.1128/Jb.187.6.2203-2208.2005. PubMed PMID: WOS:000227575100038.

6. Pester M, Bittner N, Deevong P, Wagner M, Loy A. A 'rare biosphere' microorganism contributes to sulfate reduction in a peatland. Isme Journal. 2010;4(12):1591-602. doi: 10.1038/ismej.2010.75. PubMed PMID: WOS:000285844700010.

7. Pelikan C, Herbold CW, Hausmann B, Muller AL, Pester M, Loy A. Diversity analysis of sulfite- and sulfate-reducing microorganisms by multiplex dsrA and dsrB amplicon sequencing using new primers and mock community-optimized bioinformatics. Environmental Microbiology. 2016;18(9):2994-3009. doi: 10.1111/1462-2920.13139. PubMed PMID: WOS:000387550700019.

8. Lever MA, Rouxel O, Alt JC, Shimizu N, Ono SH, Coggon RM, et al. Evidence for Microbial Carbon and Sulfur Cycling in Deeply Buried Ridge Flank Basalt. Science. 2013;339(6125):1305-8. doi: 10.1126/science.1229240. PubMed PMID: WOS:000316053400034.
